# Curative Surgery After Neoadjuvant Chemotherapy for Locally Advanced Sigmoid Colon Cancer With Extensive Abdominal Wall Invasion: A Case Report

**DOI:** 10.7759/cureus.67444

**Published:** 2024-08-21

**Authors:** Hirofumi Suzumura, Toshiaki Terauchi, Seigo Yukisawa, Masaru Kimata, Hiroharu Shinozaki

**Affiliations:** 1 Department of Surgery, Saiseikai Utsunomiya Hospital, Tochigi, JPN; 2 Department of Medical Oncology, Saiseikai Utsunomiya Hospital, Tochigi, JPN

**Keywords:** neoadjuvant chemotherapy (nact), sigmoid colon, colon cancer, abdominal wall invasion, folfox

## Abstract

Locally advanced colon cancer (LACC) can be cured under an appropriate treatment strategy, but the decision on the treatment strategy is also important in terms of long-term prognosis. In cases with extensive abdominal wall involvement, it is especially important to secure adequate margins and repair abdominal wall defects. Recently, neoadjuvant chemotherapy (NAC) for LACC has shown promise in improving the chance of cure with tumor shrinkage. Herein, we report a case of curative surgery after NAC for locally advanced sigmoid colon cancer with extensive abdominal wall invasion. A 50-year-old woman visited our hospital with anemia and an abdominal mass. The diagnosis was LACC of the sigmoid colon with abdominal wall invasion (maximum size, 12 cm), and the clinical stage was stage IIIc (T4b[skin]N1bM0). Resection of the involved skin was expected to cause an extensive abdominal wall defect. At first, a colostomy was performed, followed by NAC with leucovorin, 5-fluorouracil, and oxaliplatin (FOLFOX). Ten cycles of chemotherapy were completed without severe adverse events, and the tumor shrank in size by approximately 39%. We performed a curative sigmoidectomy combined with abdominal wall resection with adequate margins. We reconstructed the abdominal wall defect using a left anterolateral thigh skin flap. Pathological examination revealed mucinous carcinoma involving the transverse colon and abdominal wall, with luminal narrowing in the sigmoid colon. The surgical margins were negative, and the tumor was considered to have had a pathological partial response to NAC. Herein, we report a rare case of curative surgery after NAC with FOLFOX for LACC in the sigmoid colon with extensive invasion of the abdominal wall. We reconstructed the extensive abdominal wall defect with a free anterolateral thigh flap. One of the optional treatment strategies for LACC with extensive abdominal wall invasion was reported in our report.

## Introduction

In Japan, curative resection and adjuvant chemotherapy are the standard treatment for locally advanced colon cancer (LACC) without distant metastasis. In case with the tumor invades surrounding organs, extended resection is required. It is especially important to secure adequate margins and repair the abdominal wall defect in cases with extensive abdominal wall involvement. Several methods such as primary closure, component separation method, and repair with prosthetic mesh, pedicled, locoregional, or free flaps can be used for reconstructive surgery [1]. If a wide range of defects is existing, the reconstruction with a fascial graft or fasciocutaneous flap is considered.

The rate of local or metastatic recurrence after standard treatment for LACC is 15-43% [2,3]. Surgery with neoadjuvant chemotherapy (NAC) is a treatment option for LACC that addresses this issue. The expected effect of NAC on LACC is tumor shrinkage before surgery and it can provide the chance of adequate margins and complete resection [4-6].

Herein, we present a rare case of curative surgery after NAC with leucovorin, 5-fluorouracil, and oxaliplatin (FOLFOX) for LACC in the sigmoid colon existing extensive invasion of the abdominal wall. The abdominal wall was reconstructed using a left anterolateral femoral skin flap. The findings from this case would be helpful in the treatment of LACC with abdominal wall invasion.

## Case presentation

**Figure 1:**
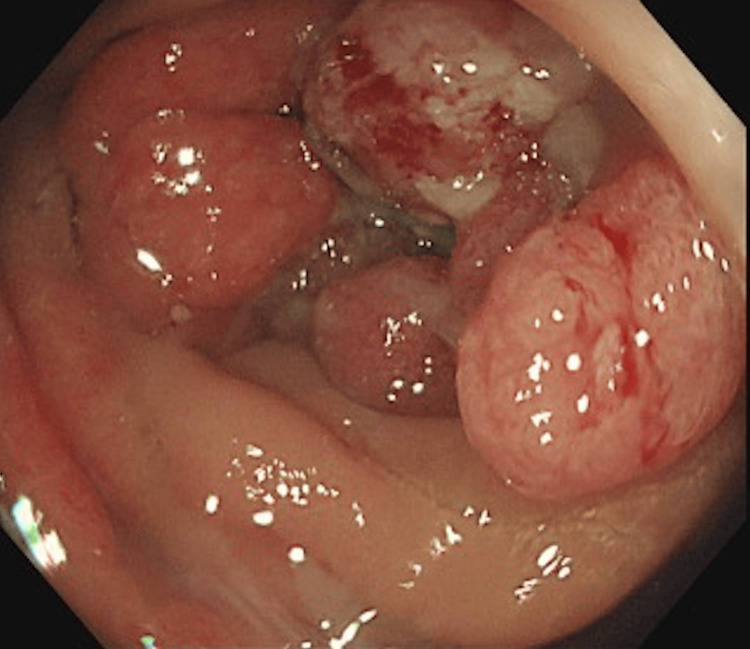
Colonoscopy findings at the initial visit A colonoscopy reveals an advanced circumferential tumor in the sigmoid colon.

A 50-year-old woman with anemia, a lower left abdominal mass, and left lower leg edema visited our hospital. The patient had no other relevant medical history. The levels of hemoglobin, D-dimer, and carcinoembryonic antigen (CEA) were 9.9 mg/dL, 5.9 μg/mL, and 10.5 ng/mL, respectively. Advanced cancer in the sigmoid colon was detected by colonoscopy, and a biopsy revealed tubular adenocarcinoma with an RAS mutation (Figure 1).

**Figure 2:**
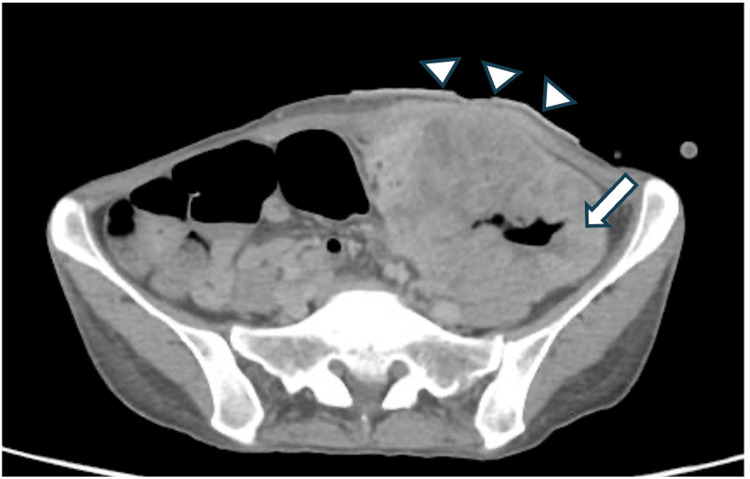
CT findings at the initial visit CT at the time of diagnosis reveals an approximately 9.7 cm tumor in a single mass in the sigmoid colon (arrow), with encapsulated air and fluid collection in the surrounding area and abscess formation. The tumor invades the subcutaneous tissues (arrowheads).

Computed tomography (CT) showed a tumor with approximately 9.7 cm diameter in the sigmoid colon, and an encapsulated air and fluid collection in the surrounding area and abscess formation was detected. The tumor invaded the abdominal wall by the subcutaneous fat (Figure 2).

Regional lymph node metastasis was suspected but distant metastases were not observed on the CT. Simultaneously, extensive thrombi were found in the bilateral pulmonary arteries, indicating pulmonary embolism (PE). Our clinical diagnosis was stage IIIc (T4b[skin]N1bM0) LACC in the sigmoid colon according to the 8th Union for International Cancer Control classification.

**Figure 3:**
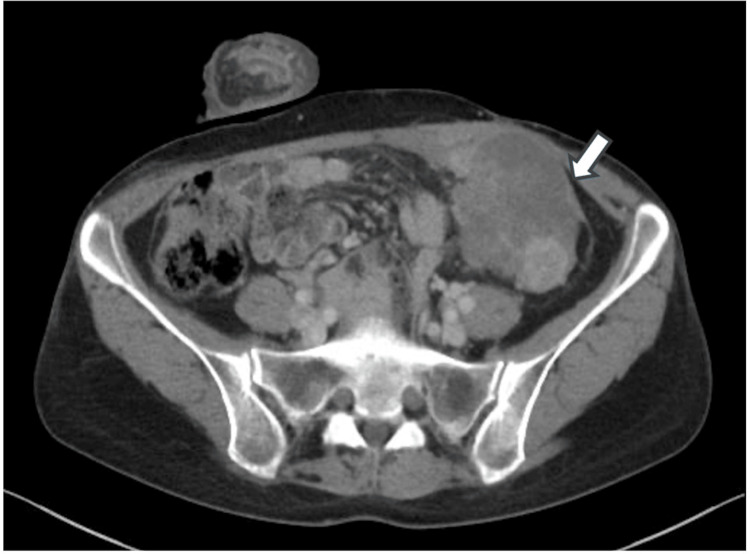
CT findings after 10 cycles of neoadjuvant chemotherapy with FOLFOX After neoadjuvant chemotherapy with FOLFOX, the tumor shrank significantly from 9.7 cm to 7 cm in diameter (arrow). Although abdominal wall invasion persisted, the area of tumor invasion was markedly reduced.

Anticoagulant induction with apixaban was initiated immediately for PE treatment. One week after the anticoagulant induction, the thrombus decreased in size and we performed a laparoscopic transverse loop colostomy. No peritoneal seeding was confirmed by the laparoscopy. We started NAC a week after the colostomy, and a FOLFOX regimen consisting of oxaliplatin, l-leucovorin, and 5-fluorouracil was selected and administered every 2 weeks. We completed 10 cycles of NAC without any severe adverse events. After NAC, the tumor shrank approximately 39% (from 7 cm × 9.7 cm to 6 cm × 7 cm) (Figure 3).

Although the abdominal wall involvement of the tumor persisted, the extent of invasion was markedly reduced. CEA levels declined from 10.5 to 5.0 mg/dL after NAC. The tumor was restaged preoperatively as T4bN1bM0. We determined that clear margins and radical resection were feasible.

**Figure 4:**
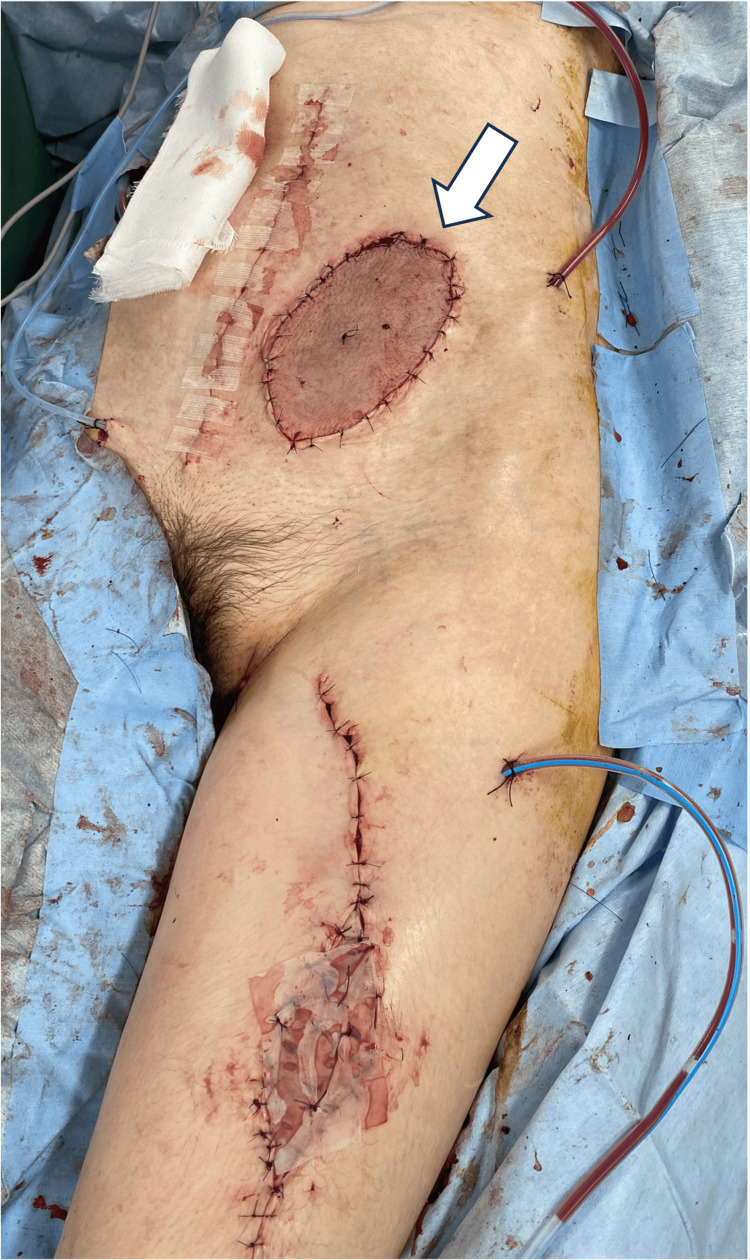
Abdominal defect after tumor resection was filled with the ALT flap ALT: anterolateral thigh

We performed an extended sigmoidectomy accompanied by abdominal wall resection with sufficient margins and stoma closure 6 weeks after the last NAC. The tumor invaded into the rectus abdominis by 3 cm from the median wound and a tumor invasion was approximately 8 cm × 12 cm. We resected rectus abdominis, transversus abdominis, and skin. The abdominal wall defect was approximately 11 cm × 16 cm long and the skin defect was approximately 8 cm × 11 cm long of the tumor resection. A pedicle anterolateral thigh (ALT) flap was created from the left thigh skin attached to the superficial thigh fascia. A continuous blood flow from the skin-perforating branch to the descending branch of the lateral femoral circumflex artery was confirmed by intraoperative ultrasonography. The superficial thigh fascia in this flap was lifted through a subcutaneous tunnel to the abdominal wall and sutured onto the left abdominal wall fascia without any tension (Figure 4).

**Figure 5:**
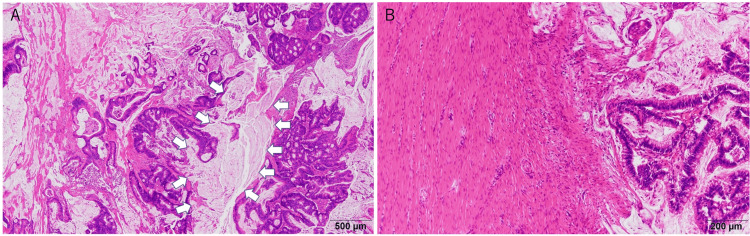
Pathological findings Pathological findings show a mucinous carcinoma in the sigmoid colon involving the transverse colon (arrow) (A) and invading the rectus abdominis sheath (B).

Pathological findings revealed a mucinous carcinoma with luminal narrowing in the sigmoid colon invading the transverse colon and anterior abdominal wall (Figures 5A-5B).

No metastases were found in the 27 lymph nodes that were dissected. The effect of NAC was a pathological partial response. Free from adenocarcinoma were found in all resected margins of the specimens. The patient was discharged on postoperative day 7 without any complications. Adjuvant chemotherapy with six courses of capecitabine with oxaliplatin (CapeOX) was administered. No recurrence has been observed 19 months after curative surgery.

## Discussion

NAC in a perioperative setting is intended to control micrometastatic cancer cells, induce tumor downstaging, and decrease long-term distant metastasis [7-9]. NAC is currently a common treatment strategy for upper gastrointestinal cancers [5,10]. Many reports showed its safety as a promising option for overcoming the poor outcomes of LACC, although NAC is not the standard treatment for colon cancer [7,11,12]. The Fluoropyrimidine, Oxaliplatin & Targeted Receptor pre-Operative Therapy for colon cancer (FOxTROT) trial reported a higher complete resection rate and a higher rate of adequate margins using NAC than adjuvant chemotherapy (96% vs. 80%) without an increase in postoperative complications [7]. On the other hand, inadequate resection margins have previously been shown to correlate with a poor prognosis in colorectal cancer [13]. Dehal et al. reported improvement in overall survival with the use of NAC in T4b patients [14]. The National Comprehensive Cancer Network (NCCN) guidelines clearly state that NAC is a possible treatment option for T4b LACC. In our case, extensive abdominal wall invasion was observed before treatment. Therefore, we selected NAC to ensure a more secure surgical margin and control systemic micrometastases.

In the present case, the tumor extended into the abdominal wall with a subcutaneous abscess formation, but accurate tumor invasion could not be detected on the surface. In the case of T4b (skin), NAC would be useful because of its direct effect on tumor shrinkage and the latency in removing infection from a subcutaneous abscess. In the present case, NAC was initiated approximately 2 weeks after the start of treatment for PE. Although there was a risk of bleeding from the tumor and complications related to the subcutaneous abscess, this seemed to be an acceptable time to initiate NAC. Furthermore, although we completed the planned chemotherapy without adverse events, the possibility of tumor progression should always be considered. There were no symptoms of bowel obstruction, and the indication for colostomy was controversial. However, infection control was essential for performing NAC. Colostomy may be useful for complete infection control, especially in preventing abscess-related events during chemotherapy.

We performed 10 cycles of FOLFOX as NAC. However, the number of cycles of NAC and the optimal regimen for LACC remain controversial. According to the NCCN guidelines, CapeOX or FOLFOX is considered an optimal regimen but the number of cycles is not mentioned. In the previous studies, three or four courses of NAC were administered to balance the efficacy of chemotherapy with NAC-related side effects and postoperative complications [7,11]. The PRODIGE 22 trial with the neoadjuvant FOLFOX 4 versus FOLFOX 4 plus cetuximab versus immediate surgery for high-risk stage II and III colon cancers showed that NAC did not improve the major pathological response rate, which was the primary endpoint of this trial, compared with adjuvant chemotherapy. The authors claimed that the limited number of NAC cycles may be responsible for this negative result [11]. In contrast, it has been reported that 15-20% of advanced colon cancers progress after 12 weeks of the same chemotherapy regimen [15,16]. In our case, we performed surgery after 10 cycles of chemotherapy, because of the extensive abdominal wall invasion at the initial diagnosis to secure a reliable margin. As a result, the tumor size was reduced by approximately 39% compared to that before NAC and a reliable R0 resection could be performed. The number of NAC cycles should be determined on a case-by-case basis.

The additional effect of adding molecular targeted agents to NAC is an interesting issue. Vascular endothelial growth factor (VEGF) inhibitors may be considered in patients with RAS mutations, but previous studies have shown negative results [17]. The neoadjuvant CAPOX (capecitabine plus oxaliplatin) and bevacizumab alone for locally advanced rectal cancer (N-SOG 03) phase II trial, utilizing CapeOX with bevacizumab (Bmab) as NAC for locally advanced rectal cancer yielded positive outcomes with respect to short-term results with completing of the regimen and pathological complete response rates. In this trial, an anastomotic leakage rate of 27.8% was observed which could be attributed to the use of Bmab in NAC [17]. Furthermore, the addition of Bmab to oxaliplatin-based regimens (i.e., CapeOX and FOLFOX) as adjuvant chemotherapy for colorectal cancer has been reported to have negative results and is not recommended [18], suggesting that VEGF inhibitors are not expected to have additional effects as NAC for colorectal cancer. However, as NAC and adjuvant chemotherapy for colorectal cancer have different targets and purposes, it may be difficult to make blanket statements.

The ALT flap was first reported in 1984 by Song et al. as a free flap for head and neck burn contractures [19]. This is a new pedicle flap concept based on the lateral femoral circumflex system. Synthetic mesh is an option to consider for abdominal wall defects that are difficult to close, but only for clean surgery. In such cases, myocutaneous flap reconstruction is necessary. In our case, the abdominal wall invasion of the tumor was resected with sufficient margins, resulting in an extensive abdominal wall defect. For such extensive defects, the ALT flap is useful for minimizing donor-site morbidity and securing a large skin flap and fascia with long vascular pedicles [20]. The defect was located near the midline of the abdomen, but a single continuous skin-perforating branch to the descending branch of the lateral femoral circumflex artery was identified, which could be used to elevate the flap in our case. The abdominal wall was reconstructed using a pedicle ALT flap. The ALT flap may be a useful reconstruction method for LACC with extensive abdominal wall involvement.

## Conclusions

Herein, we report a rare case of curative surgery after NAC with FOLFOX for LACC in the sigmoid colon with extensive invasion of the abdominal wall. An extensive abdominal wall defect was reconstructed with an ALT pedicle flap. Although the treatment strategy for LACC with extensive abdominal wall involvement is controversial, the number of NAC cycles and tumor shrinkage after NAC would be important factors for favorable outcomes. At this point, it is unclear whether this treatment strategy will improve the long-term prognosis in our case, but our experience demonstrates NAC followed by surgery with an ALT reconstruction could be a beneficial treatment option for LACC with abdominal wall involvement.
